# Limited impact of Greenland meltwater on abruptness and reversibility of future Atlantic overturning changes

**DOI:** 10.1126/sciadv.aed2633

**Published:** 2026-06-19

**Authors:** Oliver Mehling, Katinka Bellomo, Federico Fabiano, Marion Devilliers, Michele Petrini, Susanna Corti, Jost von Hardenberg

**Affiliations:** ^1^ Department of Environment, Land and Infrastructure Engineering, Politecnico di Torino, 10129 Turin, Italy.; ^2^ Institute for Marine and Atmospheric research Utrecht, Department of Physics, Utrecht University, 3584 CC Utrecht, Netherlands.; ^3^ Department of Geosciences, University of Padova, 35131 Padua, Italy.; ^4^ Institute of Atmospheric Sciences and Climate, Consiglio Nazionale delle Ricerche, 40129 Bologna, Italy.; ^5^ Danish Meteorological Institute, 2100 Copenhagen, Denmark.; ^6^ NORCE Norwegian Research Centre, Bjerknes Centre for Climate Research, 5007 Bergen, Norway.; ^7^ Institute of Atmospheric Sciences and Climate, Consiglio Nazionale delle Ricerche, 10133 Turin, Italy.

## Abstract

All climate models project that the Atlantic Meridional Overturning Circulation (AMOC) will weaken in the 21st century, but most models neglect increasing runoff from the Greenland ice sheet. Greenland meltwater is expected to exacerbate AMOC weakening, and omitting it increases the uncertainty in assessing the possibility of an abrupt AMOC collapse or tipping. Here, we test the abruptness and reversibility of AMOC changes under strong future global warming in a state-of-the-art climate model with and without high-end but physically plausible Greenland meltwater forcing. In this model, Greenland meltwater significantly exacerbates future AMOC weakening especially after 2100, but the AMOC changes until 2300 are neither abrupt nor irreversible on centennial timescales, even with added meltwater. While accounting for Greenland meltwater will increase the accuracy of climate projections, our results do not suggest a major role of Greenland meltwater for assessing the risk of future AMOC tipping.

## Teaser

Climate model simulations show the Atlantic overturning circulation can recover even from strong future Greenland ice sheet melt.

## INTRODUCTION

The Atlantic Meridional Overturning Circulation (AMOC) has a prominent role in shaping Earth’s mean climate and climate change (*1*–*4*). Climate model simulations have demonstrated that an AMOC weakening is expected to cause near-global climate impacts (*5*–*9*). Therefore, the possibility that global warming could induce an abrupt collapse or tipping of the AMOC, defined here as “a critical threshold beyond which a system reorganizes, often abruptly and/or irreversibly” following the Intergovernmental Panel on Climate Change (IPCC) definition (*10*), has been debated for several decades (*11*–*13*). Early warning indicators suggest that the AMOC is currently undergoing destabilization compatible with approaching a tipping point (*14*–*16*), although recent work has also identified new mechanisms that are expected to stabilize the AMOC (*17*, *18*). In addition, the ability of the AMOC to weaken abruptly and irreversibly with respect to (idealized) freshwater forcing has now been demonstrated across the hierarchy of ocean and climate models (*16*, *19*–*21*).

Nevertheless, while there is consensus among climate models that the AMOC will weaken until 2100 (*22*), the IPCC Sixth Assessment Report (AR6) found that none of the Coupled Model Intercomparison Project phase 6 (CMIP6) models show an abrupt AMOC collapse during the 21st century (*23*). In contrast to earlier assessments, however, the confidence in this finding was downgraded from “high confidence” to “medium confidence” in AR6 (*23*), implying that a possible collapse of the AMOC is still under debate, especially for the time horizon after 2100 (*23*–*25*). Besides model biases that could influence AMOC stability (*26*, *27*), one reason cited by the IPCC why current-generation models may not be able to simulate an AMOC collapse is that they neglect meltwater influx from the Greenland Ice Sheet (GrIS) (*23*), which is expected to accelerate with global warming (*28*).

Previous studies have shown that including realistic GrIS meltwater in future emission scenarios can exacerbate future AMOC weakening (*29*–*36*). A multimodel comparison of meltwater effects on the AMOC until 2300 concluded that including Greenland melt “significantly increases AMOC weakening [...] as well as the probabilities of an AMOC collapse under continued high greenhouse-gas emissions” (*34*). However, it currently remains an open question whether these meltwater-induced AMOC changes are associated with tipping point characteristics such as abruptness and irreversibility. In addition, the physical mechanisms of how Greenland meltwater affects the AMOC beyond 2100 have, to our knowledge, not yet been explored.

Here, we compare the AMOC response until 2300 with and without Greenland meltwater forcing with a focus on abruptness and irreversibility, as well as physical mechanisms, in model experiments carried out with the current-generation (CMIP6) climate model EC-Earth3 (*37*). The experimental setup is improved compared to previous modeling studies (*29*–*36*) by combining three aspects: We use a model with one of the higher grid resolutions in CMIP6, several ensemble members to reliably separate the meltwater effect from internal variability, and high-end but physically plausible meltwater forcing from a coupled climate-ice sheet model. EC-Earth3 is also one of the few CMIP6 models that simulates both a realistic present-day AMOC strength and negative value of freshwater import into the South Atlantic ($F_{\text{ovS}}$; Materials and Methods), whose sign is consistent with observations implying that the model is not expected to be biased toward a monostable AMOC regime (*27*). Despite a significant impact of Greenland meltwater on AMOC weakening especially after 2100, we do not find an abrupt or irreversible AMOC collapse, suggesting that the omission of Greenland mass loss in climate models does not have a major impact on the assessment of potential future AMOC tipping based on these models.

## RESULTS

### Meltwater-induced AMOC weakening

**Fig. 1.**
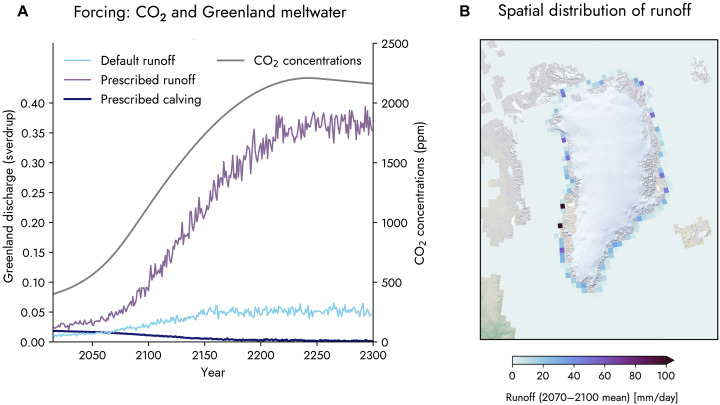
Greenland meltwater and CO_2_ forcing. (**A**) Left axis: Annual mean runoff and calving used as input for the “meltwater” simulations and runoff in the “reference” simulations for comparison. Right axis: CO_2_ concentrations in the SSP5-8.5 scenario. (**B**) Example spatial distribution of runoff (averaged over 2070 to 2100) with nonuniform input at coastal grid points.

To reliably isolate and quantify the meltwater signal, we perform two four-member initial condition ensembles with and without Greenland meltwater forcing (“meltwater” and “reference”, respectively). We prescribe Greenland runoff and calving from a state-of-the-art (CMIP6) coupled climate-ice sheet model simulation using the Community Earth System Model version 2 (CESM2)–Community Ice Sheet Model version 2 (CISM2) (*38*) under a high-end greenhouse gas emission scenario (SSP5-8.5) until 2300 (Fig. 1A and Materials and Methods). Using ice sheet model output forced by a different climate model comes with some caveats (Materials and Methods) such as inconsistent global sea level changes (which are therefore not analyzed here), but we do not expect that this affects the conclusions regarding the AMOC. The advantage of using output from CESM2-CISM2 is that Greenland meltwater forcing includes climate-ice sheet feedbacks. Greenland meltwater reaches 0.09 sverdrups by 2100 and more than 0.3 sverdrups by 2300 (Fig. 1A), which we consider a high-end but physically plausible estimate. The meltwater is routed in a physically consistent way, nonuniformly in space and time, to coastal grid points around Greenland (Fig. 1B). This reflects emerging evidence that there can be regional differences in how Greenland meltwater affects the AMOC (*39*, *40*).

**Fig. 2.**
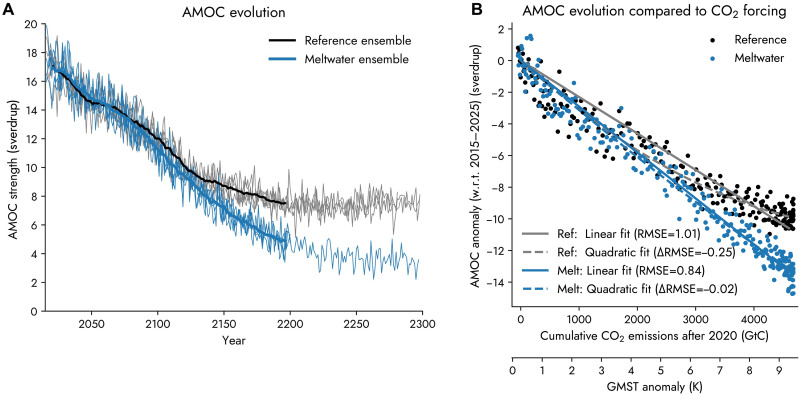
AMOC projections until 2300 with and without Greenland meltwater. (**A**) Annual mean AMOC time series for individual ensemble members (thin lines) and 10-year running mean of ensemble means until 2200 (thick lines; as the full 4 × 2–member ensemble covers the period of 2016 to 2200). (**B**) AMOC anomalies in one pair of ensemble members against cumulative CO_2_ emissions since 2015. Here, and if not mentioned otherwise, the AMOC (in depth space) is defined as the depth maximum of the overturning stream function at 26°N. w.r.t., with respect to; RMSE, root mean square error; GtC, gigatonnes of carbon.

We find that Greenland meltwater consistently exacerbates the weakening of the AMOC at 26.5°N in response to global warming (Fig. 2A). However, until 2100, the size of the effect is projected to be relatively small. For the reference ensemble without Greenland meltwater, the AMOC weakening under SSP5-8.5 is approximately linear during the 21st century and ranges between 3.8 and 7.3 sverdrups per century for different ensemble members compared to 4.5 to 8.4 sverdrups per century in the meltwater ensemble. This enhanced weakening of, on average, 0.9 sverdrups per century is consistent for all pairs of ensemble members (fig. S1A) and statistically significant (P=0.02 using a paired Student’s *t* test), although on the same order as the standard deviation of trends because of low-frequency variability (1.5 sverdrups per century in the reference ensemble).

We quantify when this significantly stronger AMOC weakening trend emerges in the meltwater ensemble compared to the reference ensemble using bootstrap resampling of the interannual to decadal AMOC variability (Materials and Methods). For the linear AMOC trend, a statistically significant difference emerges in 2092 [2086 to 2098, 66% confidence interval (CI); Fig. 2B]. Similarly, an emergence in 2100 (2091 to 2112, 66% CI) is found when the 15-year running mean of the AMOC index is used instead of the AMOC trend. This is at most a few decades after first differences between forcing scenarios emerge, given that the AMOC in CMIP6 models weakens almost independently of the scenario “until about 2060” (*23*).

Beyond the 21st century, the meltwater-induced AMOC weakening becomes stronger, in line with the strong increase in Greenland melting after 2100 (Fig. 1) due to the sustained high carbon dioxide (CO_2_) emissions in the SSP5-8.5 scenario. By the end of the 22nd century (2180 to 2200), an additional AMOC weakening of 2.5 ± 0.2 sverdrups is attributable to Greenland meltwater. We extend one member per ensemble until the end of the 23rd century, by which the AMOC stabilizes at 7.5 sverdrups in the reference simulation but weakens further to about 3.5 sverdrups with added Greenland meltwater. In summary, Greenland meltwater induces an additional AMOC weakening on the order of 10 to 20% at the end of the 21st century and up to 40% at the end of the 23rd century compared to the AMOC weakening induced by CO_2_ and consequent atmosphere-ocean feedbacks, which are accounted for in current climate models.

Even after experiencing strong meltwater input, a basin-wide Atlantic overturning cell remains in all simulations regardless of whether depth or density coordinates are used, although it is shallower and weaker in the meltwater simulation (fig. S2). This differs from entirely collapsed or reversed Atlantic overturning states previously identified in some climate models (*41*–*43*). Nevertheless, the AMOC in the meltwater simulations is on the threshold of being classified as “collapsed” using typical threshold definitions [e.g., 80% weakening compared to the preindustrial AMOC strength (*44*)]. AMOC characteristics such as northward heat transport due to overturning into the South Atlantic (fig. S3A) or shared outcropping isopycnals between the Northern Hemisphere and the Southern Ocean (fig. S3, B and C) (*45*) remain even at the end of the 23rd century, but they are strongly reduced with meltwater input. It should also be noted that the Atlantic ocean heat transport (OHT) due to overturning is close to zero at some tropical latitudes in the meltwater simulation.

We do not find signs of an abrupt AMOC change following the classical definition of a nonlinear response exceeding the rate of external forcing (*46*–*48*). As shown in Fig. 2B, the AMOC strength in the meltwater ensemble scales linearly with cumulative CO_2_ emissions (and global mean temperature) until emissions reach zero in 2250. This scaling is sublinear in the reference ensemble because of the AMOC stabilization in the 23rd century, in agreement with previous modeling studies that did not account for GrIS melt (*49*). To summarize, while it is ambiguous whether the AMOC can be classified as “collapsed,” the weakening is not abrupt.

### Shift of AMOC source regions shapes the response to meltwater

**Fig. 3.**
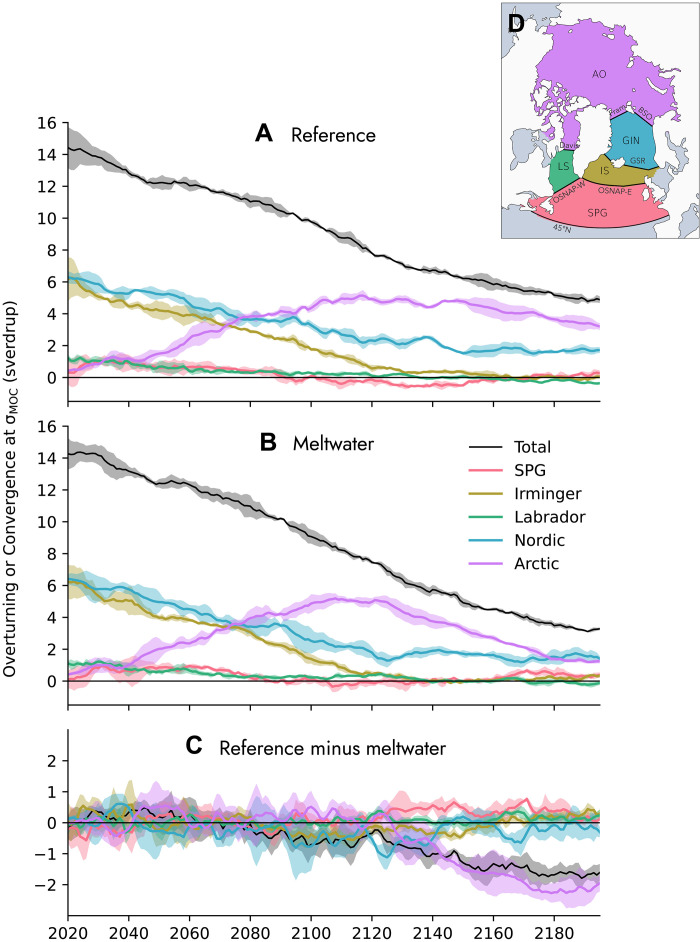
Change in AMOC source regions. Maximum overturning at 45°N (black line) and convergence at $\sigma_{\text{MOC}}$ by region: (**A**) Reference ensemble, (**B**) meltwater ensemble, and (**C**) difference between the two ensembles. All lines are for 2015 to 2200 and are smoothed with a 10-year running mean; shadings indicate the ensemble standard deviation. The inset (**D**) shows the definition of the source regions and the gateways bounding them.

The significant additional AMOC weakening after around 2100 calls for a better understanding of associated mechanisms, which have not been analyzed in previous modeling efforts (*34*). To this end, we decompose the Atlantic overturning at 45°N and its meltwater-induced weakening into the contributions of different source regions north of 45°N (defined in Fig. 3D) on the basis of the density-space overturning at the northern and southern gateways bounding these regions (Materials and Methods).

In a present-day climate, the main AMOC source regions in EC-Earth3 are the Irminger and Nordic Seas, consistent with observations (Materials and Methods) that show that the overturning is much stronger in the eastern subpolar North Atlantic than in the western subpolar North Atlantic (*50*). Both ensembles agree that the importance of the Irminger and Nordic Seas will decrease through the 21st and 22nd centuries. In turn, the AMOC is characterized by a strong northward shift of source regions in response to global warming (*51*). The Arctic Ocean, whose present-day direct contribution to the AMOC at 45°N is negligible, becomes the most important AMOC source region in EC-Earth3 by 2100 (Fig. 3, A and B). This increased role of the Arctic Ocean is partly because the overturning strength across the Arctic gateways intensifies but also because the overturning peak in density space moves more rapidly to lighter densities in the Arctic Ocean than at subpolar latitudes (fig. S4), potentially due to Arctic Ocean amplification (*52*). Consequently, the density of maximum overturning at the Arctic gateways coincides with that at 45°N ($\sigma_{\text{MOC}}$) during much of the 22nd century, whereas it is located at the larger densities of the overflow waters during the present day, contributing to the overturning further south only indirectly (e.g., via mixing at the Greenland–Scotland Ridge).

**Fig. 4.**
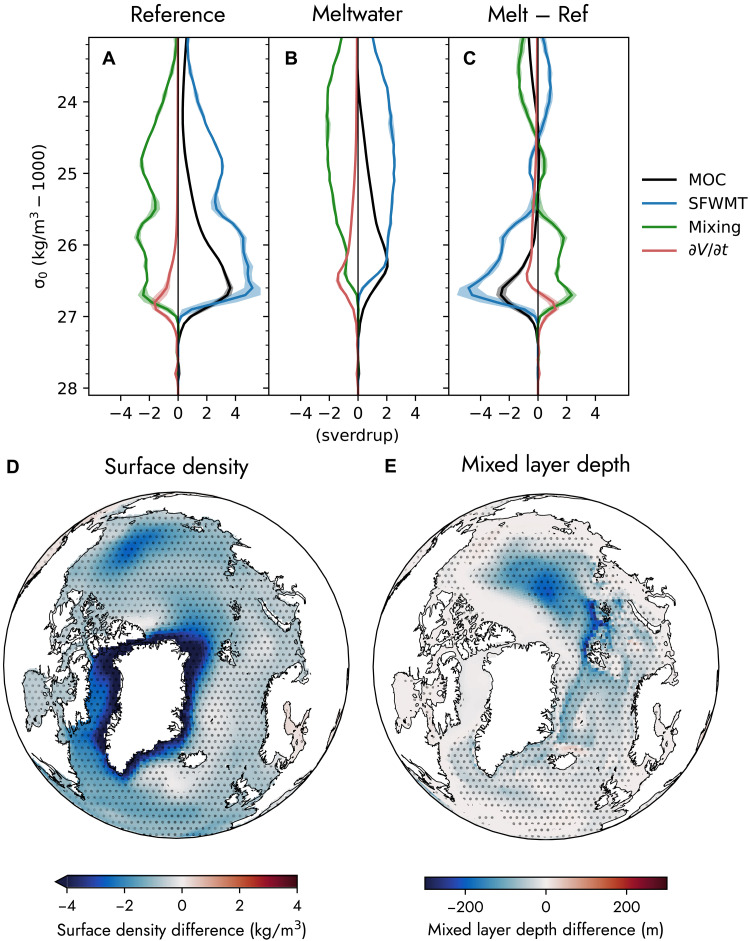
Effect of Greenland meltwater on an ice-free Arctic Ocean. (**A** to **C**) Volume and buoyancy budget [Materials and Methods; (*111*)] for the Arctic Ocean at the end of the 22nd century (A) for the reference simulation and (B) with Greenland meltwater. (C) shows the difference between (B) and (A). The overturning across the Arctic gateways (MOC) is decomposed into SFWMT, volume changes (∂V/∂t), and mixing, which is calculated as a residual. (**D** and **E**) Ensemble mean differences (meltwater minus reference) at the end of the 22nd century: (D) annual mean surface density and (E) winter (March) mixed layer depth. Areas with significant differences (P<0.05) using a *t* test are stippled.

In line with the northward shift of AMOC source regions, the Arctic Ocean contributes the most to the meltwater-induced additional AMOC weakening in the 22nd century in EC-Earth3 (Fig. 3C). This meltwater-driven weakening can be understood in terms of a volume and buoyancy budget for the Arctic Ocean (Fig. 4, A to C, and Materials and Methods). The overturning across the Arctic gateways weakens mostly because of a decrease in surface-forced water mass transformation (SFWMT), while the magnitude of interior mixing also decreases with meltwater input. Decreased SFWMT is linked to a decrease in both surface density (Fig. 4D) and surface buoyancy flux. While the former is a direct consequence of the meltwater input freshening the surface layers, the latter (as well as the smaller interior mixing component) is linked to a decrease in Arctic mixed layer depth (Fig. 4E). This is again a consequence of the lower surface density, which increases stratification. To summarize, Greenland meltwater influences the northward-shifted AMOC source regions both directly (via decreased surface density) and indirectly (via increased stratification that leads to less mixing and less heat loss).

### Reversibility of meltwater-induced AMOC changes

The emergence of a significant meltwater signal on the AMOC after continued global warming and ice sheet melt raises the question of how this meltwater input affects the reversibility of the AMOC. Previous studies using idealized “overshoot” experiments have shown that the AMOC can fully recover after CO_2_ concentrations are reversed back to their initial levels (*53*–*55*). In the CMIP6 ensemble, full AMOC recovery after an idealized ramp-up and ramp-down of CO_2_ concentrations at 1% per year (Materials and Methods) was found in seven of eight models (fig. S6) (*56*) in the 1pctCO2-cdr simulations of the Carbon Dioxide Removal Model Intercomparison Project (CDRMIP) (*57*). However, none of these previous model experiments assessed the role of Greenland mass loss in this context.

Here, we probe how the large Greenland meltwater input affects AMOC reversibility under CO_2_ reversal using a similar approach to the CDRMIP experiments. Branching off from the SSP5-8.5 scenario in 2250, CO_2_ concentrations are ramped down at 1% per year for 170 years until they reach 2015 levels and held constant thereafter. This ramp-down experiment is initialized from both the meltwater and reference experiments to isolate differences in reversibility behavior due to the previous meltwater forcing. Because no CO_2_ ramp-down is available with the ice sheet model, we also switch off the additional meltwater forcing after 2250 for these CO_2_ reversal experiments. (Ir)reversibility is most commonly defined with respect to a timescale (*10*). Here, we follow the definition in (*58*) by considering centennial timescales “after all emissions cease” (i.e., after the end of the ramp-down). This is consistent with the notion of reversibility that is assessed through more realistic, policy-relevant overshoot scenarios [e.g., (*59*, *60*)].

**Fig. 5.**
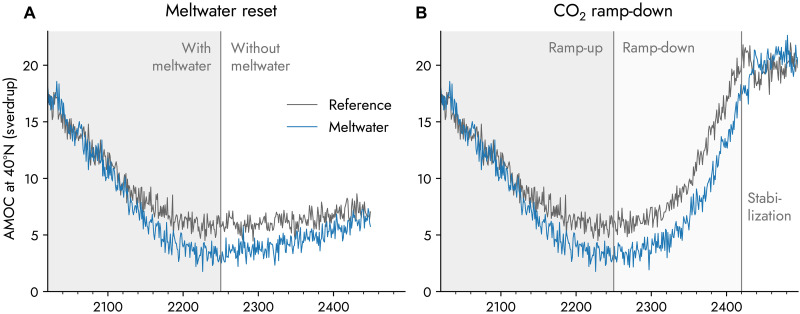
AMOC reversibility in EC-Earth3. Annual mean AMOC maximum at 40°N for (**A**) the “meltwater reset” experiments and (**B**) the “CO_2_ ramp-down” experiments (see the text for experiment setups). The dark gray–shaded time spans in both subplots are identical to the standard “reference” and “meltwater” simulations, with the meltwater forcing stopped at 2250.

The CO_2_ ramp-down experiments show the reversibility of both the greenhouse gas– and meltwater-induced AMOC changes on multidecadal and longer timescales (Fig. 5B). During the ramp-down, the AMOC remains about 2 to 3 sverdrups weaker with prior meltwater forcing, corresponding to a difference in recovery time to the same AMOC strength of, on average, 25 years. The main regional driver of AMOC differences shifts from the Arctic Ocean to the Irminger Sea as the ramp-down progresses (fig. S5), demonstrating that the meltwater effect on the AMOC recovery is linked to several source regions and not only the Arctic Ocean. After the stabilization at 2015 CO_2_ levels, both simulations reach a similar stabilized AMOC strength of around 20 sverdrups within 50 years. We conclude that prior Greenland meltwater input does not affect the AMOC reversibility under a strong CO_2_ overshoot in EC-Earth3.

There is evidence that this behavior could generalize to the CMIP6 ensemble, meaning that the AMOC recovery observed in the CO_2_ ramp-up/ramp-down CDRMIP experiments (fig. S6) would still hold if these simulations included Greenland melt. This is based on an analysis of the surface buoyancy flux averaged over 40°N to 65°N in the North Atlantic ($B_{\text{flux}}$; Materials and Methods), which has previously been proposed as an indicator for an AMOC collapse under global warming (*25*, *61*). The underlying idea is that a change from losing to gaining buoyancy over the subpolar North Atlantic might be associated with a destabilization of the AMOC (*61*, *62*).

At the end of the stabilization phase after the CO_2_ ramp-down, all models in which the AMOC recovers have returned to a $B_{\text{flux}}<0$ as in the initial state, while the only model in which the AMOC does not recover has a $B_{\text{flux}}>0$ (fig. S6). These results are consistent with the idea that stabilization below or above $B_{\text{flux}}=0$ may separate a recovering AMOC from a nonrecovering AMOC, in line with the indicator proposed by van Westen *et al.* (*61*). In EC-Earth3, the effect of the previous Greenland meltwater input is, when averaged over the ramp-down and stabilization phases, $\Delta B_{\text{flux}}=0.21\times 10^{-8}\;J\;{\text{kg}}^{-1}\;s^{-1}$. Of the six analyzed models in which the AMOC recovers after a CO_2_ ramp-down without meltwater, only one (UKESM1-0-LL) lies within this $\Delta B_{\text{flux}}$ of a zero surface buoyancy flux. Therefore, if the meltwater effect on the $B_{\text{flux}}$ indicator in EC-Earth3 is representative, Greenland meltwater of comparable effect size would be unlikely to “tip the balance” against recovery in most of the analyzed CDRMIP models.

Last, we also test whether Greenland meltwater input could have induced a transition to a different stable AMOC state under sustained high CO_2_ forcing in EC-Earth3, i.e., we test AMOC reversibility due to Greenland meltwater alone. This is more in line with an equilibrium-focused definition of irreversibility; for example, the Global Tipping Points Report defines irreversibility as a “change in a system that is not reversed under the same boundary conditions that triggered it or that takes significantly longer to recover from than the time it took to reach” (*48*). In this set of experiments (“meltwater reset”), the meltwater forcing is switched off in 2250, and CO_2_ concentrations are kept constant after 2300. In parallel, the SSP5-8.5 simulation without meltwater input is also extended under constant CO_2_ concentrations after 2300. A caveat is that these experiments approach but do not reach equilibrium; therefore, we cannot exclude millennial-scale hysteresis behavior.

Resetting the meltwater forcing under late-23rd century conditions leads to a gradual recovery of the AMOC (Fig. 5A). After 200 years, the meltwater-induced AMOC anomaly at 40°N has decreased from −2.7 to −0.7 sverdrups compared to the reference simulation. The larger recovery rate in the meltwater simulation suggests that both simulations would eventually converge to the same equilibrium. The timescale of recovery is (multi)centennial, but it does not take “significantly longer to recover from than the time it took to reach” in the spirit of the Global Tipping Points Report definition, as meltwater forcing had been applied over more than 200 years. Therefore, it appears that Greenland meltwater forcing, despite its large magnitude, also does not induce an irreversible shift to a different equilibrium state of the AMOC under very high CO_2_ concentrations in this model.

## DISCUSSION

In this study, we used an ensemble of future projections with a state-of-the-art climate model to probe the response of the future AMOC to high-end but physically plausible Greenland meltwater input. In contrast to a nonsignificant effect during the historical period (*63*), we found a small but significant additional meltwater-induced AMOC weakening of about 1 sverdrup until 2100 (about 10% of the CO_2_-induced weakening) and up to 4 sverdrups until 2300 (nearly 40% of the CO_2_-induced weakening) under very strong forcing. These numbers are in good agreement with previous studies using parametrized Greenland meltwater input (*33*, *34*). As climate projections beyond the 21st century are becoming more widely used, the increasing importance of Greenland meltwater for AMOC weakening after 2100 emphasizes the need to incorporate realistic meltwater estimates into these long-term projections.

However, we found that the combined CO_2_- and meltwater-induced AMOC weakening in our model was neither abrupt (with respect to external forcing) nor irreversible (on centennial timescales), characteristics often associated with climate tipping points (*10*, *13*). Instead, with Greenland meltwater, the AMOC weakening scaled linearly with cumulative CO_2_ emissions and global mean temperature. This linearity is an important subject for future study: If corroborated by other climate models, it might also yield additional insights into the physics of future AMOC weakening, such as why the AMOC strength diverges relatively late in different emission scenarios (*22*).

The results presented here are based on a single climate model and should be interpreted as such. One aspect that could be model-dependent is that most of the meltwater-induced AMOC weakening in this study is via the Arctic Ocean. The importance of the Arctic Ocean in EC-Earth3 is consistent with previous work demonstrating a northward shift of AMOC source regions (*51*, *64*) and areas of deep convection (*65*–*67*) into the Arctic Ocean as the winter sea ice edge retreats northward under global warming. However, only a relatively small number of CMIP6 models (including variants of EC-Earth) show sustained deep convection in the Arctic Ocean at the end of the 21st century (*68*). To our knowledge, there are currently no established observational constraints that would rule in favor or against sustained dense-water formation in the Arctic after 2100, and EC-Earth3 is not an outlier within the CMIP6 ensemble regarding present-day Arctic Ocean salinity and temperature biases (*69*). In models with a less sustained Arctic contribution to the AMOC, or more generally, a more strongly weakened AMOC due to global warming alone (*25*), we expect that the meltwater effect on the AMOC would be smaller than the one found in EC-Earth3. While potentially model-dependent, the novel mechanism described in this study raises the possibility that the Arctic Ocean could become an important mediator between the GrIS and the AMOC under strong global warming.

The AMOC reversibility experiments were also performed with a single model, but the realistic AMOC strength and a negative $F_{\text{ovS}}$ as in observations increase our confidence that EC-Earth3 is not biased toward a monostable AMOC regime (*27*). While the AMOC in EC-Earth3 recovers after “hosing” in a preindustrial background climate (*70*), this behavior is not uncommon in CMIP6 models, as half of the participating models in (*70*) exhibited similar behavior. Recently, AMOC recovery after Greenland meltwater input and a CO_2_ ramp-down has independently been noted in CESM2 (*71*), one of the models in which the AMOC does not recover after hosing (*70*). Our multimodel analysis of surface buoyancy flux changes after an idealized CO_2_ reversal indicated that most CMIP6 models recover to a North Atlantic state with sufficient buoyancy loss for which Greenland meltwater is not expected to “tip the balance” against AMOC recovery.

Nevertheless, the absence of abruptness and irreversibility even under strong melt rates of more than 0.3 sverdrups may seem at odds with the robustness of a freshwater-induced AMOC collapse across the model hierarchy (*20*, *43*, *72*). Besides a potential impact of model biases, there are several possible explanations. First, even if the AMOC is in a bistable regime under preindustrial conditions, it is possible that the AMOC shifts outside the bistable regime at very high CO_2_ concentrations and only a single, weak AMOC stable state remains or that the basin of attraction of an “Off” shrinks considerably. This possibility calls for a better (conceptual) understanding of the interplay of CO_2_ and freshwater forcing on the AMOC (*12*, *73*), especially under transient forcing. A related gap in understanding is how timescale-dependent effects relate to (or hinder) potential AMOC tipping under transient forcing. For example, under strong CO_2_ forcing, the AMOC in climate models usually remains in a weak state on multicentennial timescales but often recovers on (multi)millennial timescales (*74*). The presence of chaotic transients can also lead to delayed tipping on multicentennial or longer timescales (*75*–*78*), which may not be captured on the timescales simulated here.

Last, it has previously been shown that the same amount of North Atlantic freshwater input has a reduced effect on the AMOC under global warming due to changes in ocean stratification and in the North Atlantic gyre structure (*79*). A corollary is that strong meltwater forcing under a moderate CO_2_ increase might provide a higher risk for AMOC tipping. However, Greenland melt rates also generally scale with atmospheric temperatures and, therefore, CO_2_ concentrations (*33*). This creates a tug-of-war between an increasing amount of meltwater discharge and the weakening ocean sensitivity to meltwater input under global warming, although this relationship could break down on (multi)millennial timescales if the GrIS itself crosses a tipping point (*80*, *81*).

In addition to comparing the response in different climate models, future studies should assess whether the overturning changes shown here are robust in high-resolution ocean models, which resolve mesoscale eddies [e.g., (*82*)]. Nevertheless, (*83*) demonstrated that the magnitude of AMOC weakening due to Greenland meltwater does not depend strongly on the ocean resolution despite different meltwater propagation pathways, increasing our confidence in estimates of meltwater-induced AMOC weakening from CMIP6-class models. Last, given that the uncertainties regarding the impact of Antarctic meltwater on the AMOC are considerable (*82*, *84*, *85*), simulations assessing the concurrent impacts of Greenland and Antarctic meltwater on the AMOC in long-term scenarios are needed in the future.

## MATERIALS AND METHODS

### Model and AMOC characteristics

We perform model experiments with a state-of-the-art coupled climate model, EC-Earth3 (*37*), which participated in CMIP6 (*86*). EC-Earth3 consists of the atmospheric model IFS cy36r4, the land-surface scheme H-TESSEL (*87*), the ocean model NEMO 3.6 (*88*), the sea ice model LIM3 (*89*), and the OASIS3-MCT coupler (*90*). EC-Earth3 is run at its standard resolution for CMIP6, i.e., a horizontal resolution of about 80 km (TL255) for the atmosphere and 1° (about 100 km) for the ocean, with a grid refinement to 1/3° in the tropical ocean. In the vertical, 91 levels are used for the atmosphere and 75 levels for the ocean, where layer depths range from 1 m near the surface to 200 m in the deep ocean.

EC-Earth3 successfully reproduces the most important basin-scale AMOC metrics at the beginning of the scenario runs (2016 to 2022). At 26°N, the AMOC strength is 17.1 ± 0.9 sverdrups, in agreement with the observational value of 16.9 ± 1.2 sverdrups from the RAPID array (*91*). In the subpolar North Atlantic, the simulated overturning across the OSNAP-East line is 15.2 ± 0.8 sverdrups, again in good agreement with observations [16.3 ± 0.6 sverdrups (*92*)]. While EC-Earth3 does not capture the overturning across the OSNAP-West line, this contribution is small (around 3 sverdrups) in observations such that the total subpolar overturning of 12.5 ± 0.09 sverdrups in EC-Earth3 is only slightly weaker than observed [16.7 ± 0.6 sverdrups (*92*)]. An important metric for AMOC stability, the freshwater transport due to overturning at 34°S ($F_{\text{ovS}}$), is −0.014 ± 0.010 sverdrups in EC-Earth3. This is slightly larger than in observations [−0.16 ± 0.09 sverdrups (*93*)] but has the correct sign (negative as observed) often associated with the AMOC being in a bistable regime (*12*). Hence, EC-Earth3 is one the very few CMIP6 models that both simulate a realistic AMOC strength and the correct sign of $F_{\text{ovS}}$ (*27*).

### Experiment setup

We conduct two ensembles (“reference” and “meltwater”) of future projections under the SSP5-8.5 scenario (*94*), the strongest global warming scenario considered by the IPCC Sixth Assessment Report with unabated greenhouse gas emissions beyond the 21st century (*95*). This scenario can be regarded as a “worst-case, no-policy” scenario suitable to study extreme climate outcomes (*96*). In SSP5-8.5, CO_2_ concentrations reach more than 1000 parts per million (ppm) (about 4× preindustrial) by the end of the 21st century and more than 2000 ppm (about 8× pre-industrial) by 2200, stabilizing at similarly high levels afterward (*95*). In EC-Earth3, this leads to a global mean surface temperature increase of 5.5, 10.1, and 10.9 K compared to preindustrial at the end of the 21st, 22nd, and 23rd centuries, respectively.

The “reference” experiments use the standard CMIP6 version of EC-Earth3, except for fixing the surface albedo at the location of present-day ice sheets to 0.8 (the default ice sheet albedo in EC-Earth). This change was implemented because IFS “parametrizes” ice sheets as a 10-m snow pack and exposes low-albedo bedrock after it melts, inducing an unrealistically strong temperature feedback. In comparison, a fixed ice sheet albedo is more in line with the moderate albedo changes even under prolonged strong CO_2_ forcing (*97*) and enables studying the effect of Greenland meltwater alone, leaving the opportunity for future model experiments to study the effect of changes in the ice cover separately. The “meltwater” experiments use the same EC-Earth3 configuration as the “reference” runs, except for prescribing runoff and calving from the GrIS, which is described in the following section.

We use an ensemble of simulations initialized from different initial conditions to separate forced changes from internal variability. The initial conditions are sourced from a subset of the 50-member Swedish Meteorological and Hydrological Institute (SMHI) Large Ensemble with EC-Earth3 (*98*). Because low-frequency variability—and, therefore, ensemble spread—in EC-Earth3 is dominated by the AMOC (*37*), we select four ensemble members that span the range of simulated present-day AMOC strength and projected 21st century AMOC weakening under SSP5-8.5 (fig. S7A). This includes the two members with the strongest and least pronounced weakening as well as two members with average AMOC characteristics, yielding an overall representative sample (fig. S7B). Both four-member ensembles (“reference” and “meltwater”) are initialized from the same set of initial conditions at the start of 2016. Note that because of the slight modification to the model, our reference simulations sample a different realization of internal variability compared to the SMHI Large Ensemble members so that the rate of weakening is not expected to be identical. However, the strong correlation between the initial AMOC state and 21st century AMOC weakening (fig. S7A) means that we still expect to sample a wide range of rates of AMOC weakening.

### Meltwater forcing

In the “meltwater” ensemble, we prescribe runoff and calving from a fully coupled climate-ice sheet model, CESM2 coupled to CISM2 for the GrIS (*97*). CESM2 (*99*) has a nominal resolution of 1° for atmosphere and ocean, and CISM2 (*100*) was run at a 4-km resolution. Ice-sheet runoff is routed following topography gradients to the nearest ocean grid point, while calving is spread diffusively over a radius of up to 300 km from the coast to mimic icebergs (*38*). Climate projections under the SSP5-8.5 scenario until 2100 were presented in (*38*).

We prescribe CESM fields for ocean surface fluxes from runoff (monthly) and calving (annually) from 2016 until 2300, including the diffusive spreading of calving. We set grid cells with negative river runoff values, which are artifacts from water conservation in CESM’s land model, to zero such that the total runoff into the ocean matches the runoff from the ice-sheet model well. These fluxes were remapped conservatively onto the native NEMO grid before being passed to EC-Earth3 following the implementation in (*101*). At coupling time, we set internally calculated runoff and calving over Greenland to zero to avoid double counting. As in the standard EC-Earth3 configuration, runoff is inserted into the ocean at sea surface temperature and zero salinity and through a depth of up to 150 m to prevent physical and numerical issues from injecting large amounts of runoff into the topmost ocean cell (*88*). In the meltwater ensemble, the runoff depth mask is updated around Greenland to match the runoff climatology by the end of the 23rd century. For calving, we account for the latent heat flux of melting.

The area-integrated time series of runoff over Greenland is shown in Fig. 1A. During the historical period, both the fully coupled ice sheet model in CESM2-CISM2 (19.4 millisverdrups; all values averaged 1981 to 2010) and the simple mass balance approach in EC-Earth (8.6 millisverdrups) produce a realistic magnitude of runoff compared to observations [13.2 millisverdrups; (*102*)]. In the future projections, GrIS runoff strongly increases in CESM2-CISM2, reaching 0.09 sverdrups by the end of the 21st century and more than 0.3 sverdrups by the end of the 23rd century (Fig. 1A). In the EC-Earth reference simulations, Greenland runoff levels off at about 0.07 sverdrups after 2150, a similar value as in many other CMIP6 models. The meltwater forcing prescribed in the model intercomparison of Bakker *et al.* (*34*) was also about 0.07 sverdrups in 2300 such that no significant effect on the AMOC would be expected with their meltwater parametrization. Calving in CISM2 decreases during the 21st century and beyond (Fig. 1A) (*38*), but the uncertainty of this projection can be considered large because CISM2 does not account for ocean forcing at marine-terminating Greenland margins [compare (*103*)]. In any case, the contribution of calving in SSP5-8.5 is expected to be small compared to the strongly increasing runoff.

We note that there are several limitations to our approach. First, we prescribe monthly fields from a single model such that the meltwater trajectory inherits the boundary conditions from the CESM2 simulation. A recent study comparing long-term projections from a standalone GrIS model forced with boundary conditions from different general circulation models showed that CESM forcing produced the largest GrIS mass loss (*104*), implying that the meltwater forcing derived from CESM2-CISM2 is likely at the upper end of the CMIP6 range. Second, our implementation does not account for the differences in surface mass balance between CESM2 and EC-Earth3 such that the sea level rise in the EC-Earth3 “meltwater” runs is different from the integrated CISM2 mass loss. However, we do not analyze sea level metrics here, making this an acceptable trade-off over ad hoc corrections. We preferred the forcing approach from a fully coupled model over parametrizations [e.g., (*33*, *34*, *105*)] for several reasons: The extended SSP5-8.5 forcing extends far beyond their calibration range; parametrizations typically give regional averages, while CESM2 runoff is resolved at a 1° resolution; they do not eliminate the role of model biases, as the (regional or global) climate model forcing used for calibration can substantially affect the runoff sensitivity to warming (*106*); and they also do not take into account differences in surface mass balance. As a final caveat, fixing the ice sheet geometry and albedo to present-day values allows to cleanly separate the effect of meltwater, but these changes are expected to further weaken the AMOC [e.g., (*107*, *108*)]. However, the effect is expected to be of second order compared to the effects of CO_2_ and meltwater.

### Overturning and source region diagnostics

First, we analyze the AMOC in the depth space at the conventional latitude of 26.5°N by taking the depth maximum of the annual mean overturning stream function (*109*). At subpolar latitudes, density (σ) coordinates are preferred over depth (*z*) coordinates because isopycnals tend to be strongly sloped in the east-west direction (*50*). Following (*110*), we therefore compute the overturning north of 45°N in density coordinates referenced to the surface ($\sigma_{0}$). The stream function is then defined as (*111*)

$$\Psi \left(\sigma \right)=-\int_{x_{1}}^{x_{2}}\int_{\sigma_{\text{bot}}}^{\sigma }v\left(x,\sigma^{\prime },t\right)d\sigma^{\prime }dx$$ (1)

where *v* is the northward velocity; $x_{1}$ and $x_{2}$ are the western and eastern boundaries of the basin, respectively; and $\sigma_{\text{bot}}$ is the bottom (maximum) density. Using this definition, the overturning is zero at the bottom and equal to the net volume transport for σ→0. Following previous modeling studies (*112*, *113*), we do not apply any compensation term between neighboring straits (except for the comparison against observed values above). We compute Ψ(σ) at 45°N, two sections east and west of Greenland that approximately follow the OSNAP array (*50*), the Greenland-Scotland Ridge, Davis Strait, Fram Strait, and the Barents Sea Opening (Fig. 3D). Stream functions are computed from monthly data using an adapted version of the “line method” from the StraitFlux package (*114*).

Following (*113*), we define the convergence of overturning *M* in a region as the difference between Ψ(σ) at its southern and northern boundaries. This way, the overturning across 45°N is decomposed into the sum of convergences of the five regions north of 45°N (Fig. 3D)

$$\Psi_{45N}\left(\sigma \right)=M_{\text{SPG}}\left(\sigma \right)+M_{\text{IS}}\left(\sigma \right)+M_{\text{LS}}\left(\sigma \right)+M_{\text{GIN}}\left(\sigma \right)+M_{\text{AO}}\left(\sigma \right)$$ (2)

where SPG refers to the subpolar gyre, IS refers to the Irminger Sea, LS refers to the Labrador Sea, GIN refers to the Nordic Seas, and AO refers to the Arctic Ocean and Baffin Bay.

To obtain a closed budget, we subtract the Bering Strait throughflow from $\Psi_{45N}$ and assume that the contributions from other marginal seas (Baltic Sea, Hudson Bay, etc.) are negligible, which is confirmed by the very good agreement between the left-hand and right-hand sides of Eq. 2.

Similarly to (*111*), in our analysis, we focus on the (time-dependent) density $\sigma_{\text{MOC}}$, which is defined as the density at which the overturning at 45°N is at its maximum. This can be interpreted as the density that bounds the AMOC lower limb. Evaluating Eq. 2 at $\sigma_{\text{MOC}}$ therefore quantifies the net contribution of each region to the AMOC lower limb, regardless of which processes drive diapycnal transformations.

### Volume and buoyancy budget

If a region is characterized by inflow of lighter denser water masses and outflow of denser water masses, the overturning can be related to the transformation of water masses at the surface in the so-called Walin framework (*115*). The volume and buoyancy budget in a region can be computed as (*111*)

$$F\left(\sigma \right)+G\left(\sigma \right)=\frac{\partial V\left(\sigma \right)}{\partial t}+\Psi_{S}\left(\sigma \right)-\Psi_{N}\left(\sigma \right)$$ (3)

where F(σ) is the SFWMT defined below, V(σ) is the ocean volume with a potential density larger than σ, and $\Psi_{S}\left(\sigma \right)$ and $\Psi_{S}\left(\sigma \right)$ are the overturning across the southern and northern gateways of the region, respectively, calculated using Eq. 1. For the Arctic Ocean, $\Psi_{N}\left(\sigma \right)=0$ because there is no overturning across Bering Strait. G(σ) quantifies interior mixing that cannot be diagnosed directly from climate model output and is therefore calculated as a residual (*111*).

F(σ) is given by

$$F\left(\sigma \right)=\frac{1}{\Delta \sigma }\iint_{A}\left[-\frac{\alpha }{C_{p}}\;Q+\beta \frac{S}{1-S}\Phi_{\text{FW}}\right]\;\Pi \left(x,y;\sigma \right)dx\;dy$$ (4)

where α and β are the thermal expansion and haline contraction coefficients, respectively; $C_{p}$ is the specific heat capacity of seawater; *Q* is the air-sea heat flux; *S* is the sea surface salinity; $\Phi_{\text{FW}}$ is the surface freshwater flux; and Π(x,y;σ) selects the outcrop region of a density range $\sigma \pm \frac{\Delta \sigma }{2}$. It is defined as

$$\Pi \left(x,y;\sigma \right)=\left\{\begin{array}{cc} 1 & \text{if}|\tilde{\sigma }\left(x,y\right)-\sigma |\leq \frac{\Delta \sigma }{2}\\ 0 & \text{elsewhere} \end{array}\right.$$ (5)

where $\tilde{\sigma }\left(x,y\right)$ is the potential density at the location (x,y). *F* is analogous to a stream function and is computed from monthly fields for surface density, heat, and freshwater fluxes (evaporation minus precipitation minus runoff minus sea-ice melt) and subsequently averaged into an annual mean climatology. A spacing of $\Delta \sigma =0.1\;\text{kg}\;m^{-3}$ is used for all budget terms in Eq. 3.

### Time of emergence

For any given year, the difference in ensemble means between the reference and meltwater ensembles is compared using a one-sided, paired Student’s *t* test. Here, two simulations initialized from the same initial condition but subject to different meltwater trajectories are treated as paired observations because of the long memory in the AMOC time series arising from low-frequency (centennial) variability (*116*, *117*). Given that the AMOC weakening in all ensemble members is approximately linear in until at least 2120, the *t* test is first applied to the linear AMOC trend (2016 to the specified year), and a sensitivity test is performed using 15-year running means instead of the trend. The year of emergence is then defined as the first year from which on the difference is always statically significant (P<0.05).

For an uncertainty estimate of emergence times, we repeat this procedure on an ensemble of 1000 surrogate time series for each ensemble member. To construct the surrogates, the interannual-to-decadal variability (“residuals”) is obtained by subtracting a fifth-order polynomial fit from the original AMOC time series. Then, the residuals are resampled using the “random phasing” method of Ebisuzaki (*118*), which preserves the weak autocorrelation of the residuals better than conventional bootstrapping, and added back to the polynomial fit.

### CMIP6 model data

To put the reversibility experiments from EC-Earth3 in context, we analyze multimodel output from CDRMIP (*57*) as part of CMIP6. The 1pctCO2 and 1pctCO2-cdr CDRMIP experiments use an idealized CO_2_ ramp-up/ramp-down protocol starting under preindustrial conditions. First, CO_2_ concentrations are increased by 1% per year (“ramp-up”) until they reach four times preindustrial values (around 1140 ppm) after 140 years. Subsequently, CO_2_ concentrations are decreased at 1% per year (“ramp-down”) for another 140 years until they return to preindustrial values. The simulations are then extended for a minimum of 50 years under fixed preindustrial conditions (“stabilization”). A total of eight CMIP6 models listed in Fig. 5 provided AMOC output for this experiment.

### Surface buoyancy flux indicator

The surface buoyancy flux B(x,y) is calculated from a heat flux and a freshwater flux contribution as

$$B=-\frac{g\;\alpha }{\rho_{0}C_{p}}\;Q+\frac{g\;\beta }{\rho_{0}}\frac{S}{1-S}\Phi_{\text{FW}}$$ (6)

where *g* is the gravitational acceleration, $\rho_{0}$ is the reference density of seawater, and all other quantities are defined as in Eq. 4. van Westen *et al.* (*61*) proposed that the average of *B* over the region of 40°N to 65°N in the North Atlantic, referred to as $B_{\text{flux}}$, could serve as a stability indicator for the AMOC, similarly to the approach in (*62*).

Here, this $B_{\text{flux}}$ indicator is computed on the basis of monthly EC-Earth3 or CMIP6 model output of sea surface temperature and salinity (tos and sos) and surface heat and freshwater fluxes (hfds and wfo). We verified that the conclusions remain unchanged when the average is taken over 40°N to 80°N, including the Nordic Seas. The analysis was performed for EC-Earth and seven CDRMIP models. (MIROC-ES2L did not provide sufficient heat flux output.) For some models in the CDRMIP ensemble, the surface freshwater flux was only available for the ramp-up phase, but the changes in the freshwater component in Eq. 6 are small compared to those in the heat flux component for all models. Therefore, if the freshwater variable for the ramp-down was missing, we mirrored the freshwater flux of the ramp-up phase and used the average of the first 10 years of the ramp-up for the stabilization phase.

### Ocean heat transport

Meridional OHT in a basin is calculated at each latitude *y* within NEMO and is defined as

$$\text{OHT}\left(y,t\right)=\rho_{0}C_{p}\int_{z_{\text{bot}}}^{0}\int_{x_{W}}^{x_{E}}v\left(x,y,z,t\right)\;\Theta \left(x,y,z,t\right)\;dx\;dz$$ (7)

where $\rho_{0}$ is a reference seawater density, $C_{p}$ is a reference heat capacity, *v* is the meridional velocity in m/s, and Θ is the potential temperature in °C.
