# Supplementary material for: Limited impact of Greenland meltwater on abruptness and reversibility of future Atlantic overturning changes
Source: Sci Adv. 2026 Jun 19;12(25):eaed2633. doi: 10.1126/sciadv.aed2633 (PMC13281800; doi:10.1126/sciadv.aed2633)
Supplement: Supplementary file 1 — Figs. S1 to S7 [file sciadv.aed2633_sm.pdf]

Science Advances
AAAS

Supplementary Materials for

Limited impact of Greenland meltwater on abruptness and reversibility of future Atlantic overturning changes

Oliver Mehling *et al.*

Corresponding author: Oliver Mehling, o.m.mehling@uu.nl

Sci. Adv. **12**, eaed2633 (2026)
DOI: 10.1126/sciadv.aed2633

This PDF file includes:

Figs. S1 to S7

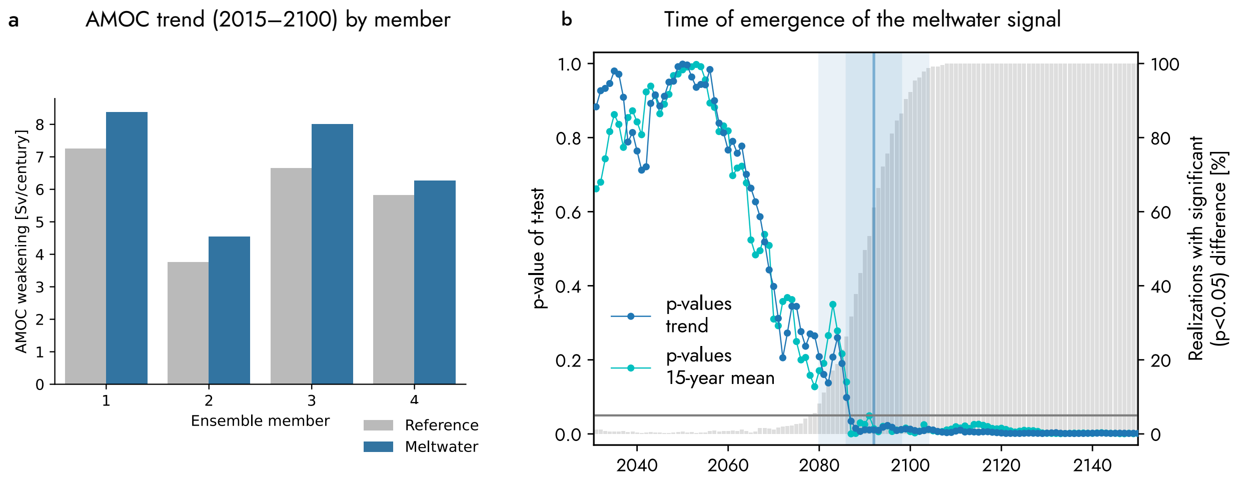

Figure S1: **21st-century AMOC weakening and emergence of the meltwater signal.** (a) Linear trends of AMOC weakening (in Sv/century) during 2015–2100 for each ensemble member. Paired bars are initialized from the same initial conditions. (b) Left axis: p-values for the one-sided t-test whether trends (blue line) or 15-year running means (cyan line) differ significantly between the reference and meltwater ensembles. The grey line shows the 0.05 threshold. Right axis: Percentage of realizations using a bootstrap test (Methods) that show a significant difference in trends between the reference and meltwater ensembles. The blue line and shadings indicate the median, 66% and 95% confidence intervals of the time of emergence (Methods).

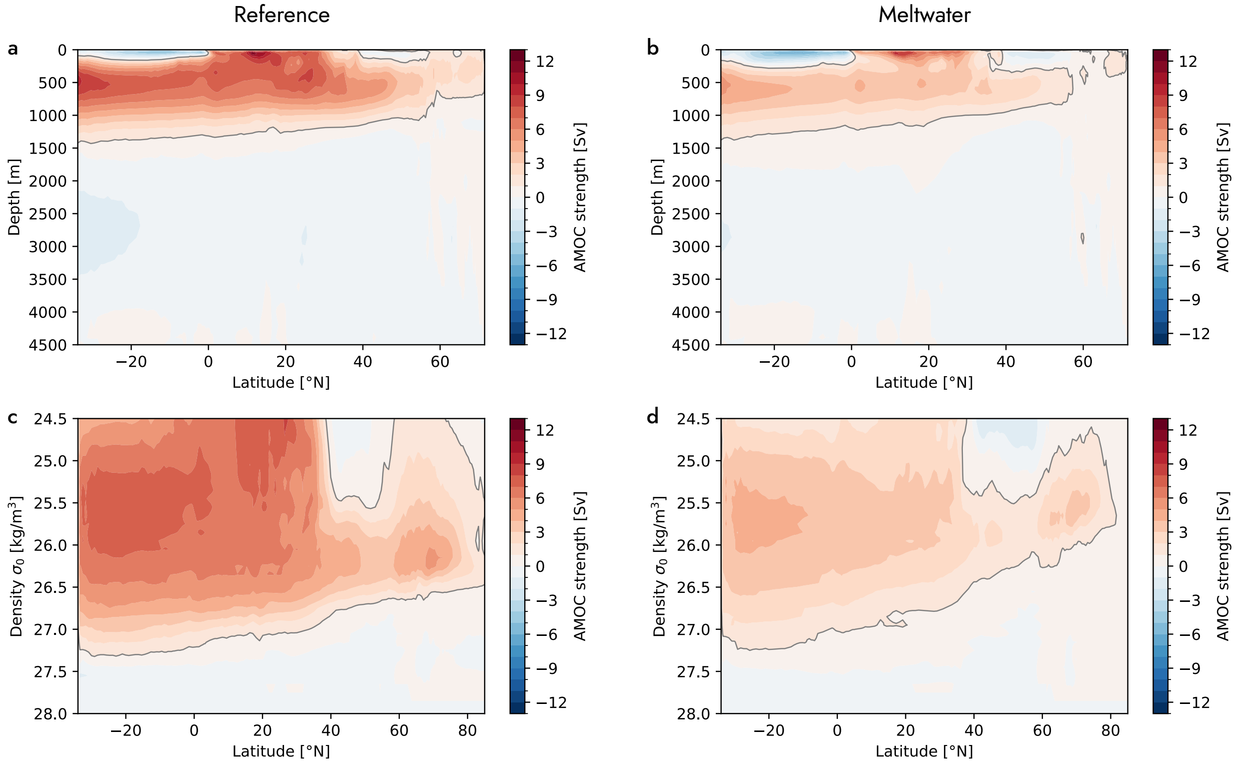

Figure S2: **AMOC streamfunctions at the end of the 23rd century.** AMOC streamfunctions in depth space (first row) and density space (second row) at the end of the simulation (2270–2300): (a, c) Reference simulation, (b, d) Meltwater simulation. Grey contours indicate the 1 Sv isolines.

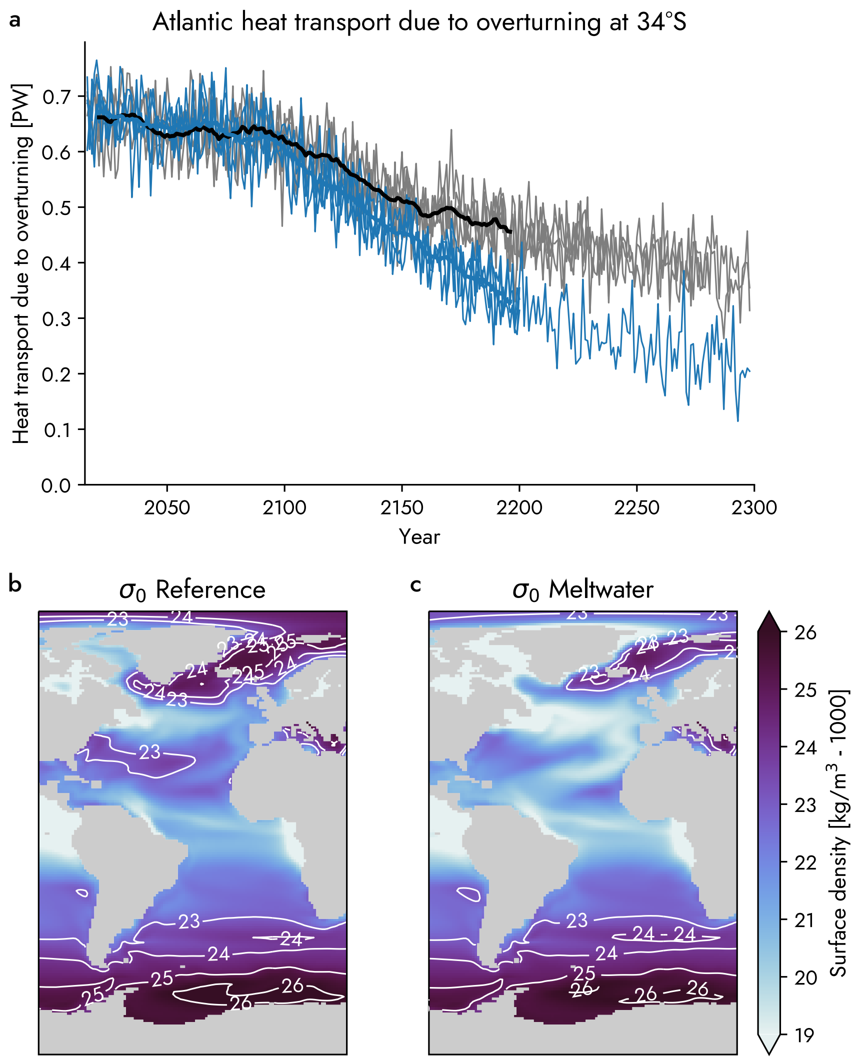

Figure S3: **Characteristics of a sustained AMOC.** (a) Time series of Atlantic heat transport due to overturning (Methods) for the reference and meltwater ensembles, (b, c) Annual mean surface density (σ_0) at the end of the 23rd century for the reference and meltwater simulations. Shared isopycnals between the North Atlantic and Southern Ocean are shown as white contours.

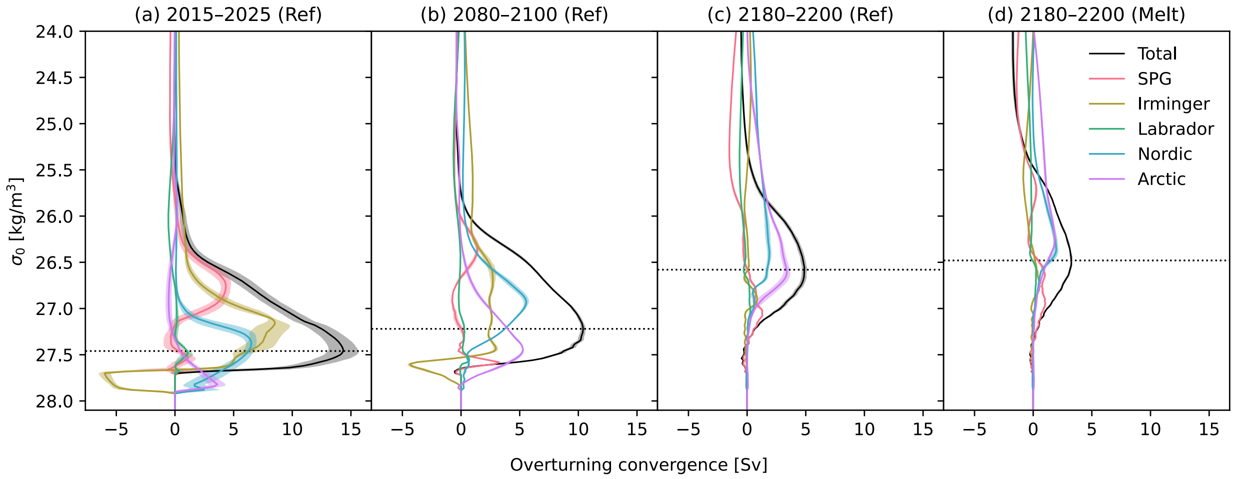

Figure S4: **Changing overturning convergence by region.** Climatologies of σ-space overturning at 45°N (black lines) and convergence by region. Shadings indicate the ensemble standard deviation. The dotted horizontal line indicates σ_{MOC}, the density at which the overturning streamfunction at 45°N is at its maximum.

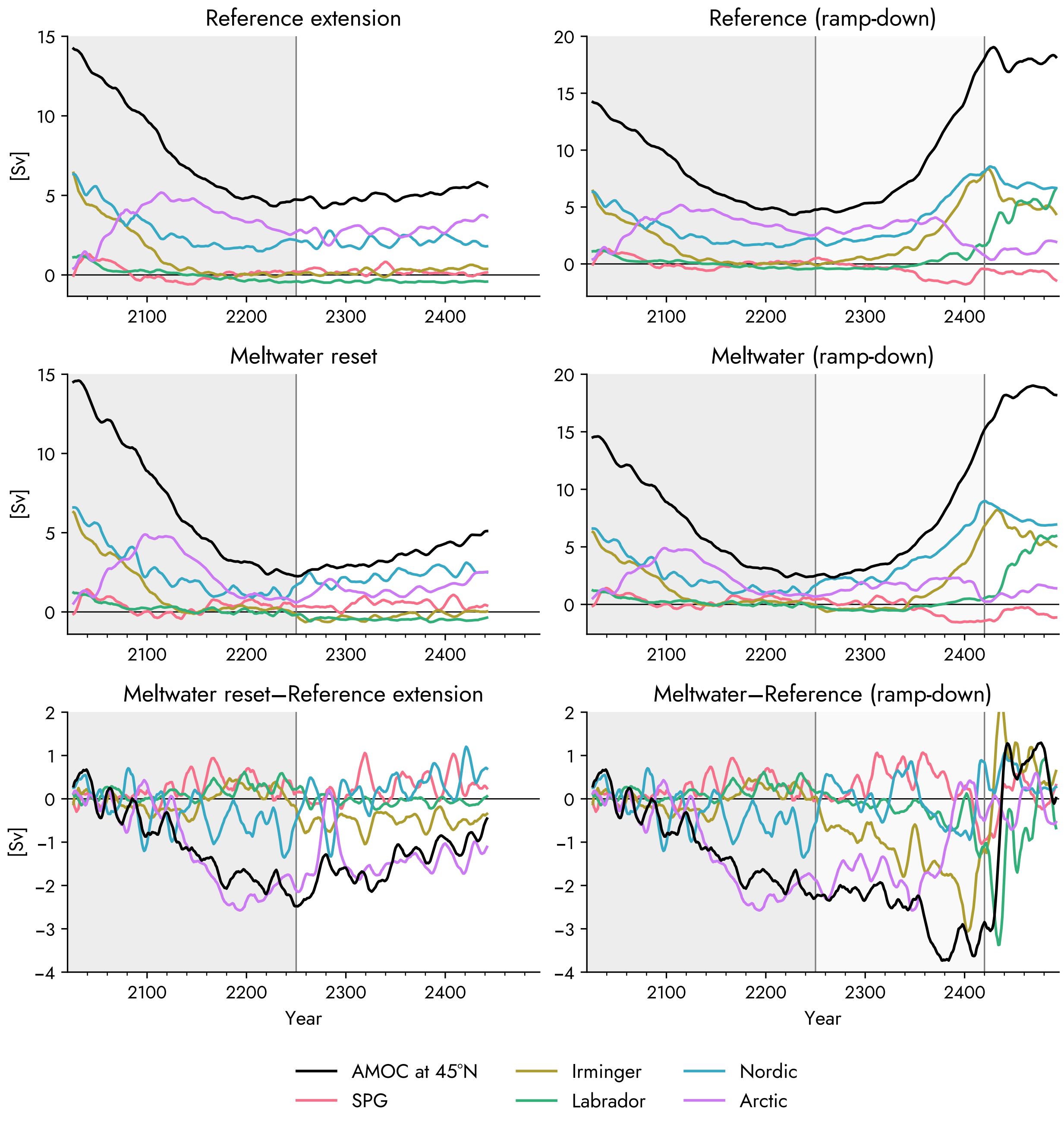

Figure S5: **Convergence at σ_{MOC} in EC-Earth3 reversibility experiments.** Same as Fig. 3, but for the reversibility experiments shown in Fig. 5.

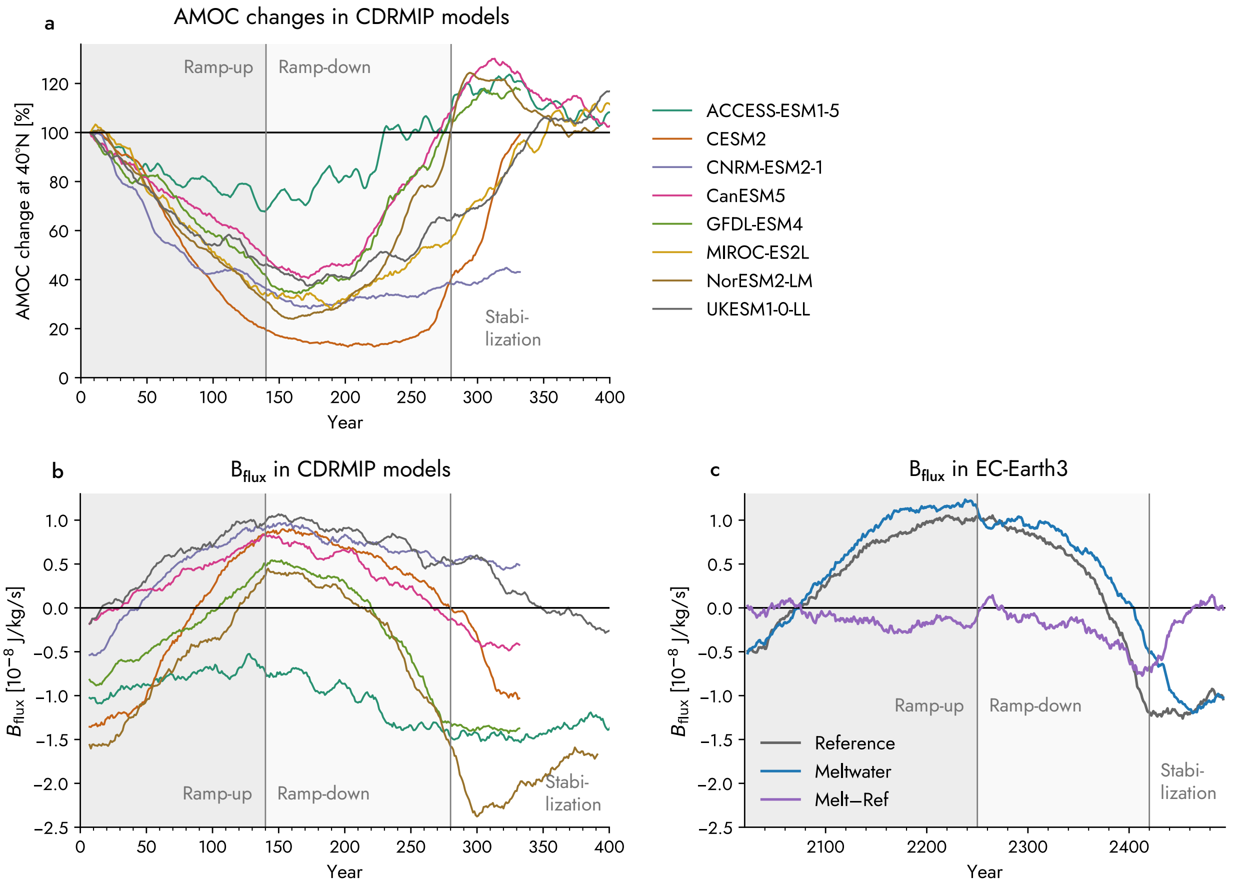

Figure S6: **AMOC and surface buoyancy flux changes in the CDRMIP ensemble.** (a) AMOC change in different CMIP6 models in the CDRMIP reversibility experiments (*57*). Starting from pre-industrial conditions, these experiments consist of a CO_2 ramp-up at 1% per year for 140 years, followed by a ramp-down at 1% per year for another 140 years and a stabilization period at pre-industrial CO_2 concentrations for at least 50 years. AMOC changes are indicated as a percentage of the initial (year 0–15) AMOC strength. (b) Surface buoyancy flux averaged over 40°N–65°N in the North Atlantic (B_{flux}) for the same CDRMIP simulations, (c) B_{flux} for the EC-Earth3 reversibility experiments with and without Greenland meltwater, with the difference between the two experiments shown in purple. All quantities in panels a–c are smoothed using a 15-year running mean.

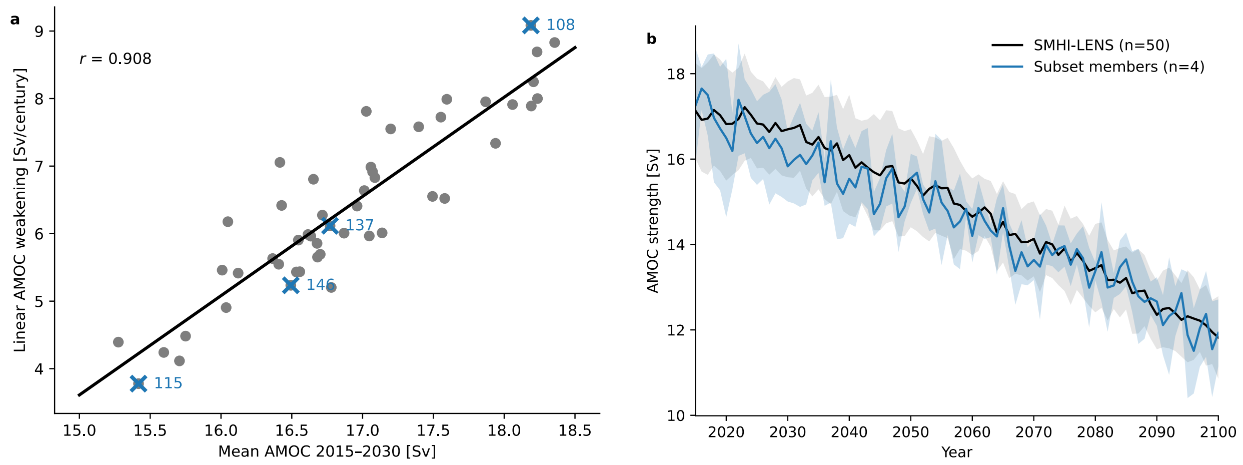

Figure S7: **Ensemble member selection strategy** based on the SMHI Large Ensemble. (a) Relation between initial (2015–2030) mean AMOC strength at 26°N and linear AMOC trend during 2015–2100 in the 50-member EC-Earth3 large ensemble (*99*) for the SSP5-8.5 scenario. The selected ensemble members and their original variant labels (rXXXi1p1f1) are highlighted with blue crosses. (b) AMOC time series (ensemble mean plus and minus one standard deviation) under SSP5-8.5 forcing for the full 50-member ensemble (black) and the 4-member subset (blue). While the subset mean is slightly lower than the ensemble mean between the 2020s and 2040s, overall the subset captures the ensemble mean and spread well, especially in the second half of the 21st century.
